# Characterization and Phylogenetic Analysis of the First Complete Chloroplast Genome of *Shizhenia pinguicula* (Orchidaceae: Orchideae)

**DOI:** 10.3390/genes15111488

**Published:** 2024-11-20

**Authors:** Yuan Chen, Yanlin Zhao, Quan Yan, Wei Wu, Qingqing Lin, Guoqiang Chen, Yanfang Zheng, Mingqing Huang, Shiming Fan, Yanxiang Lin

**Affiliations:** 1 College of Pharmacy, Fujian University of Traditional Chinese Medicine, Fuzhou 350122, China; 2220408021@fjtcm.edu.cn (Y.C.); 2230408028@fjtcm.edu.cn (Y.Z.); 2240408016@fjtcm.edu.cn (W.W.); linqq597@126.com (Q.L.); yfzheng@fjtcm.edu.cn (Y.Z.); hmq1115@126.com (M.H.); 2 College of Computer, National University of Defense Technology, Changsha 410073, China; yanquan21@nudt.edu.cn; 3 College of Ocean Food and Biological Engineering, Jiangsu Ocean University, Lianyungang 222005, China; gqchen@jou.edu.cn; 4 Department of Chinese Materia Medica, College of Pharmacy, Fujian University of Traditional Chinese Medicine, Fuzhou 350122, China

**Keywords:** Orchidaceae, Orchideae, *Shizhenia pinguicula*, chloroplast genome, phylogenetic analysis, evolution, conservation genetics

## Abstract

Background: Genomic analysis is crucial for better understanding the evolutionary history of species and for their conservation. *Shizhenia pinguicula* is a rare medicinal plant endemic to China. However, the complete chloroplast genome of this species has not been reported to date. Insufficient genomic research on *S. pinguicula* has hindered effective conservation efforts for this valuable plant. Methods: In this study, we sequenced and assembled the first complete chloroplast genome of *S. pinguicula* using Illumina sequencing technology. We conducted a comparative analysis of its chloroplast genome with related species and reconstructed phylogenetic relationships. Results: The chloroplast genome of *S. pinguicula* exhibited a typical quadripartite structure with a length of 158,658 bp. A total of 123 genes, 118 simple sequence repeats, and 51 dispersed repetitive sequences were identified. The inverted repeat boundaries were significantly expanded, along with the pseudogenization and loss of multiple NDH genes. Codon usage bias is primarily influenced by natural selection and other factors, with the *ycf3* gene under positive selection. Additionally, 10 hypervariable regions were detected for species identification and evolutionary studies. Phylogenetic analysis indicated that *Ponerorchis gracilis* and *Hemipilia yajiangensis* form a clade, with *S. pinguicula* as their sister species, located at the basal position of the *Ponerorchis*-*Hemipilia* alliance. Conclusions: The chloroplast genome structure and gene content of *S. pinguicula* exhibit certain degrees of variation compared to other species within the Orchidinae subtribe. This genome should be useful for further investigations into the biology of *Shizhenia* and the development of biodiversity conservation strategies.

## 1. Introduction

**Figure 1:**
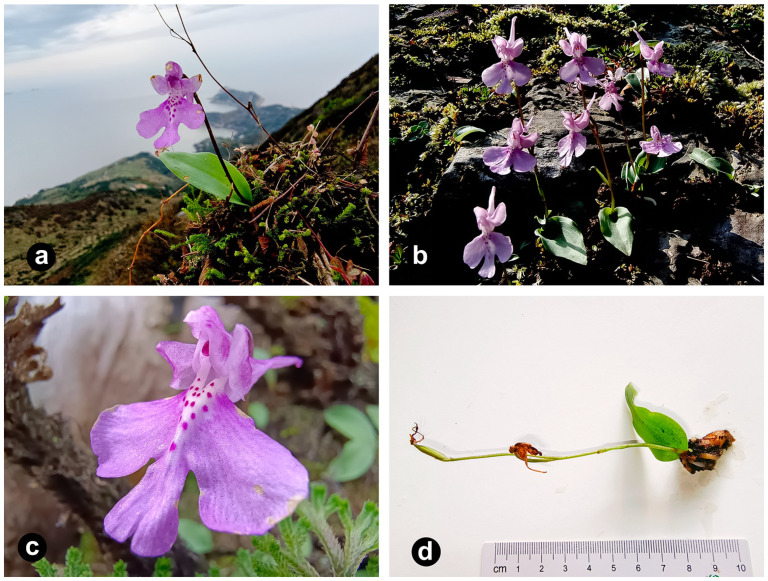
Photographs of the morphological characteristics of the *S. pinguicula* plant used in this article. (**a**) *Shizhenia pinguicula* has a preference for growing among mosses on cliffs. (**b**) The flowers of *S. pinguicula* are solitary, with a minority having two flowers. (**c**) The labellum of *S. pinguicula*’s flowers is fan-shaped, with lateral lobes which are nearly square and a middle lobe which is smaller and oval. (**d**) *Shizhenia pinguicula* possesses only a single leaf which is nearly basal.

*Shizhenia pinguicula* (Rchb. f. et S. Moore.) X. H. Jin, L. Q. Huang, W. T. Jin & X. G. Xiang (Orchidaceae) is a rare and endemic Orchideae species in China [1,2]. Jin et al. identified it as belonging to the genus *Shizhenia* in honor of the great traditional Chinese medicine expert Shi-Zhen Li [3]. This species prefers to grow on moist, rocky soils in forests or moist grasslands and in valleys at an altitude of 200–400 m (Figure 1) [4]. It is well-known for its medicinal value, with its tubers and the entire plant traditionally used for detumescence, analgesia, and activating blood circulation to dissipate blood stasis [5]. However, due to its narrow habitat range and high vulnerability to external disturbances, its population is scarce and difficult to recover from damage, making this species urgently needing effective protection.

Recent research [6] has discovered that *S. pinguicula* is one of the earliest diverging species within the East Asian clade of Orchideae s.l.. This clade encompasses a complex group, with the classification of the *Ponerorchis*-*Hemipilia* alliance standing out as a noteworthy concern. After Jin et al. [3] merged *Hemipiliopsis* Y. B. Luo & S. C. Chen into *Hemipilia*, Tang et al. [6] reconstructed the phylogenetic tree based on six markers (nuclear nrITS, low-copy *Xdh*, plastid *matK*, *psbA-trnH*, *trnL-F*, and *trnS-trnG*), merging East Asian *Amitostigma* and its alliance (including *Neottianthe* Schltr., *Ponerorchis*, *Tsaiorchis* Tang & F. T. Wang, and *Hemipiliopsis*) into *Hemipilia* s.l.. Further research expanded the sampling and, based on the results of phylogenetic analysis using seven DNA markers (*matK*, *psaB*, *rbcL*, *trnL-F*, *trnH-psbA*, and nrITS, *Xdh*), proposed recognizing five genera within the *Ponerorchis* alliance: *Hemipilia*, *Ponerorchis* s.l., *Sirindhornia*, *Shizhenia*, and *Tsaiorchis* [7]. This proposal was also supported in Chen’s study [8]. Different scholars and relevant works have significant disagreements on the definition of the genera of Orchideae from China.

Chloroplasts (cp) are the key organelles in green plants for photosynthesis, enabling the conversion of light energy into chemical energy, thereby providing the energy necessary for life activities [9]. These organelles possess DNA that is maternally inherited and independent from the nuclear genome, capable of autonomous replication. The biochemical processes within cp necessitate proteins that are encoded partly by the cp genome and partly by the nuclear genome. Furthermore, numerous nuclear genes exert influence over cp formation, showing their status as semi-autonomous organelles [10].

Generally, all the genes present in cp are collectively referred to as the cp genome. The cp genome of plants typically exhibits a double-stranded circular structure with a length ranging from 120 to 220 kb, comprising a large single-copy (LSC) region and a small single-copy (SSC) region separated by a pair of inverted repeat (IR, including IRb and IRa) regions. Characterized by its simplicity of structure, high conservation, and abundance of copies, the cp genome is ideally suited for phylogeny studies, unencumbered by issues like gene duplication or paralogous interference [11]. Owing to the continuous advancements in sequencing technologies and the reduction in costs, complete cp genome sequences have grown increasingly utilized for phylogenetic relationships both within species and across genera [12,13,14,15]. However, the phylogenetic analysis of tribe Orchideae is currently confined to nuclear nrITS and a restricted selection of plastid gene fragments [3,6,7,16]. The availability of the complete cp genome of *S. pinguicula* presents a valuable resource that can be used to identify additional molecular markers, enhance the accuracy of phylogenetic reconstructions, and establish a theoretical foundation for the conservation of this species.

Codon preference refers to the variations in codon usage frequencies among different genes within the same species. This variation is influenced by multiple factors, with selective pressure, gene mutation, and genetic drift being the primary causes of codon preference. Codon preference, to a certain extent, affects gene expression and protein translation, thereby assisting organisms in adapting to their environments at the molecular level [17]. Therefore, studying the codon usage patterns in the cp genomes of plants is beneficial for optimizing gene expression to enhance gene transformation and provide theoretical support for species conservation.

Previously, nrITS and plastid markers such as *matK*, *psbA-trnH*, *trnL-F*, and *trnS-trnG* were employed to elucidate relationships among species within the Orchideae subtribe. Unfortunately, the cp genomes of this subtribe, particularly those belonging to the East Asian clade, have not been adequately investigated. It remains uncertain whether the cp DNA of *S. pinguicula*, positioned at the base of the East Asian clade, differs from and exhibits variations compared to the cp DNA of other species within the same clade. Herein, we revealed the complete cp genome characteristics of *S. pinguicula* and performed a structural variation analysis. This contributed to deepening our understanding of *Shizhenia*’s evolutionary history and provided genomic resources for future biodiversity conservation efforts.

## 2. Materials and Methods

### 2.1. Plant Materials and DNA Extraction

A *S. pinguicula* plant was collected from Yushan Town, Fuding, Fujian, China. The voucher specimen (No. 35098220240329001LY) was preserved in the Herbarium of Fujian University of Traditional Chinese Medicine. Freshly collected tender leaves were frozen in liquid nitrogen and then transported to the laboratory, where they were stored in a −80 °C freezer. A DNA Quick Plant System (TIANGEN BIOTECH Co., Ltd., Beijing, China) was adopted to extract total genomic DNA from the young leaves.

### 2.2. DNA Sequencing, Assembly, and Annotation

The quality of the extracted DNA was assessed using agarose gel electrophoresis and a UV spectrophotometer. The total DNA was utilized to construct a library for sequencing on the Illumina NovoSeq 6000 sequencing platform (Illumina, San Diego, CA, USA). A total of 150 bp paired-end reads were generated, amounting to approximately 5.12 GB of short sequences in total. Fastqc v0.11.9 (http://www.bioinformatics.babraham.ac.uk/projects/fastqc/, accessed on 10 October 2024) was applied to evaluate the sequencing quality of all raw data. Low-quality reads and sequences with suboptimal lengths were filtered out using Fastp v0.23.4 software [18], yielding high-quality clean reads for further assembly and annotation.

Subsequently, Getorganelle v1.7.7.0 software [19] was used to assemble a complete cp circular genome. The sequence coverage and depth were plotted by a python script [20]. The annotation of the cp genome was conducted using the Geseq website [21], with the closely related species *Hemipilia yajiangensis* (NC_067080) [22] serving as reference genome. The annotation results were reviewed and manually corrected. The tRNAscan-SE v2.0 software [23] was employed to verify the annotations of tRNAs, ultimately leading to the final annotated information. The physical map of the complete cp genome was generated using the CPGView tool [24]. The complete cp genome and the final annotation of *S. pinguicula* were submitted to GenBank (PP979534).

### 2.3. Codon Usage Bias Analysis

Relative synonymous codon usage (RSCU) represents the ratio calculated by taking the usage frequency of a particular codon as the numerator and the average usage frequency of all synonymous codons encoding the same amino acid as the denominator [25,26]. RSCU values vary significantly among different species, and recent studies have shown that this value is correlated with gene expression levels [27]. The RSCU values for *S. pinguicula*, *Ponerorchis gracilis*, and *Hemipilia yajiangensis* were calculated using MEGA 7.0 software.

Neutrality plot analysis is one of the methods used to assess the factors influencing codon preference. We calculated the GC content for the first (GC1), second (GC2), and third (GC3) codon positions separately and plotted a standard curve with the GC3 of each gene on the abscissa and the GC12 (the average of the GC1 and GC2) on the ordinate.

The effective number of codons (ENC) reflects the degree of deviation from random codon usage and is widely used to measure the level of codon bias [28]. We used the Chips tool from the Emboss v6.6.0 package to compute the ENC values. A two-dimensional scatter plot (ENC-plot) was generated with the GC3 as the abscissa and the ENC as the ordinate. Each dot represents a gene. Genes located on or slightly below the standard curve indicate a strong influence of mutational pressure on codon usage bias; conversely, genes below the standard curve suggest a greater influence from selection and other factors.

The Parity Rule 2 (PR2) plot analysis is designed to avoid the imbalance of mutations among the third-position bases of codons. We retained the amino acids with four degenerate synonymous codons for calculating the base content of the third codon position. A scatter plot was then drawn with G3/(G3 + C3)|4 on the abscissa and A3/(A3 + T3)|4 on the ordinate. The degree and direction of the base bias were judged by the distance of each point from the center.

### 2.4. Repeat Sequences Analysis

The MISA v2.1 tool [29] was used to detect simple sequence repeats (SSRs) in the cp genome of *S. pinguicula*, with the minimum repeat numbers of mononucleotide to hexanucleotide set at no less than 10, 5, 4, 3, 3, and 3, respectively. The REPuter online website (https://bibiserv.cebitec.uni-bielefeld.de/reputer, accessed on 11 October 2024) [30] was utilized to analyze dispersed repeats in the cp genome of *S. pinguicula*, including forward (F), reverse (R), palindromic (P), and complementary (C) repeats. The hamming distance, maximum computed repeats, and minimal repeat size were set to 3, 5000, and 30, respectively.

### 2.5. Chloroplast Genomes Comparison

For this investigation, CPJSdraw v1.0.0 software [31] was used to compare the boundaries of the LSC, SSC, and IR regions across cp genomes from species belonging to different genera. The cp genome data were sourced from the *S. pinguicula* assembled in this study, along with five Orchidaceous species retrieved from the NCBI database (https://www.ncbi.nlm.nih.gov/, accessed on 14 October 2024), including *H. yajiangensis* (OM009241) [22], *P. gracilis* (MN200376) [32], *Ophrys insectifera* subsp. *aymoninii* (MW309825) [33], *Gymnadenia crassinervis* (MW322684), and *Galearis cyclochila* (MN200388) [32]. Utilizing the mVISTA online tool (https://genome.lbl.gov/vista/mvista/submit.shtml, accessed on 14 October 2024) [34] with *H. yajiangensis* as the reference genome, we compared the similarity among the cp genomes of these six Orchideae plants, selecting the Shuffle-LAGAN program. We also extracted both the common genes and intergenic spacer (IGS) sequences for DNA sequence polymorphisms analysis using DnaSP v5.10.01 software [35]. The result was plotted using ChiPlot (https://www.chiplot.online/, accessed on 15 October 2024).

### 2.6. Selective Pressure Analysis

Selective pressure refers to the external forces acting upon the process of biological evolution, altering the direction of this process, and enabling the organism to better adapt to its environment [35]. Based on the YN model, we used the KaKs_Calculator v3.0 program [36] to calculate the nonsynonymous substitution rate (Ka), synonymous substitution rate (Ks), and their ratio (Ka/Ks) for the CDS genes of *S. pinguicula*, *H. yajiangensis*, and *P. gracilis*. When Ka/Ks is less than 1, indicating that Ks has a higher value, synonymous substitutions are dominant, and amino acid functions are preserved. Conversely, if Ka/Ks is greater than 1, Ka is dominant, suggesting that nonsynonymous substitutions are prevalent, and natural selection exerts positive selection on amino acids. Genes with Ka/Ks ≥ 45 or NA indicate that there are almost no nonsynonymous sites in the gene, and therefore, they were not considered in our analysis.

We also conducted branch-site analysis specifically for *S. pinguicula*, *H. yajiangensis*, and *P. gracilis* using the CODEML tool of PAML v4.9 software [37]. We designated *S. pinguicula* as the foreground branch and selected two models: the null branch-site model (model = 2; NSsites = 2; fix_omega = 1; omega = 1) and the alternative branch-site model (model = 2; NSsites = 2; fix_omega = 0; omega = 2) with reference to Fang’s research [38]. Subsequently, the likelihood ratio test (LRT) and Bayes empirical Bayes (BEB) analysis were employed to examine sites under positive selection [39]. The *p*-value was calculated using the chi-square tool in PAML v4.9 [37]. Sites were considered to be under positive selection when the *p*-value of the LRT was <0.05 and the BEB value was >0.95. Finally, the secondary structure of proteins containing these positively selected sites was predicted using the PSIPRED website (http://globin.bio.warwick.ac.uk/psipred/, accessed on 14 October 2024) [40] and the SWISS-MODEL online tool (https://swissmodel.expasy.org/, accessed on 14 October 2024) [41].

### 2.7. Phylogenetic Analysis

We constructed phylogenetic trees using the complete cp genome sequence of *S. pinguicula* and 28 closely related Orchideae species, including 10 from *Habenaria* [32,42,43], 5 from *Ophrys* [33,44,45], 3 from *Dactylorhiza* [32,46], 3 from *Platanthera* [32,47], 2 from *Pecteilis*, and 1 each from *Galearis*, *Gymnadenia*, *Hemipilia*, *Ponerorchis*, and *Satyrium* [48]. The outgroups were *Cyclopogon longibracteatus* (MN597436) [49], *Goodyera schlechtendaliana* (KT886431), and *Spiranthes sinensis* (MK936427) [50] from the tribe Cranichideae. The cp genomes mentioned above were aligned using MAFFT v7.520 software [51], followed by the filtering of the aligned sequences with the R package alignmentFilter v1.0.0 [52]. Subsequently, phylogenetic trees were constructed employing the maximum likelihood (ML), Bayesian inference (BI), and maximum parsimony (MP) methods. For the ML tree construction, IQ-Tree v2.0.3 software [53] was used to obtain the optimal tree-building model, which was identified as GTR + F + I + G4, and then utilized to construct the ML tree with a bootstrap value set to 1000. When constructing the BI tree with MrBayes v3.2.7a software [54], the optimal model detected by the IQ-Tree v2.0.3 software was selected. The MCMC algorithm was run for 1,000,000 generations, sampling every 100 generations, and a burn-in of 25% was applied to discard initial trees, ultimately yielding a tree annotated with posterior probabilities. The MP tree was constructed using PAUP v4.0b software [55], with a bootstrap value set at 1000.

## 3. Results

### 3.1. Chloroplast Genomes Features

**Figure 2:**
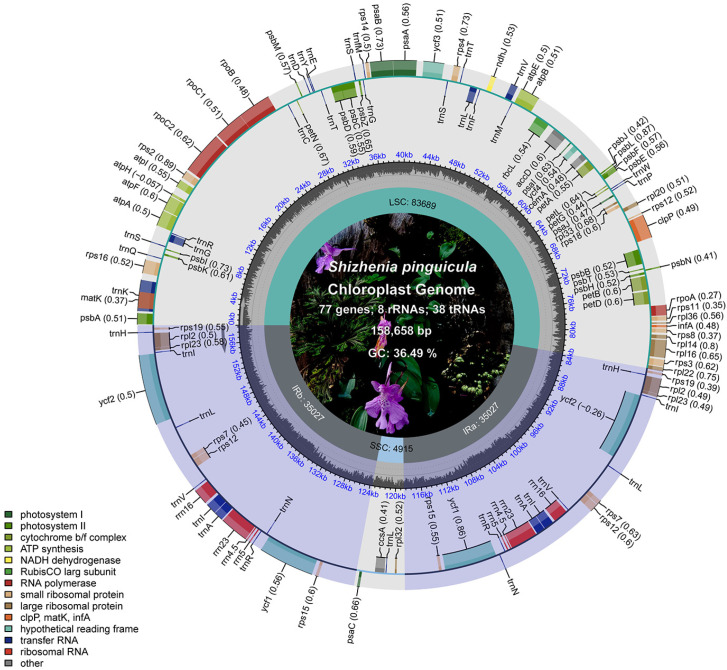
Physical map of the cp genome in *S. pinguicula*. The gray column chart in the inner circle represents the GC content. The inner and outer sides of the outer circle represent the genes in clockwise and counterclockwise directions, respectively. Different color blocks represent gene groups with different functions, and the functions corresponding to the colors are annotated in the lower left corner.

The cp genome of *S. pinguicula* achieved a 100% coverage (Figure S1) and exhibited a closed circular double-stranded structure, adhering to the classic quadripartite genome structure (Figure 2). The total length of the genome was 158,658 bp, with the LSC and SSC regions measuring 83,689 bp and 4915 bp, respectively, while the two IR regions were each of 35,027 bp in length. Nucleotide composition analysis showed an overall guanine–cytosine (GC) content of 36.49% and a adenine–thymine (AT) content of 63.51%. Within the LSC, the GC and AT contents were 34.20% and 65.80%, respectively, whereas in the IR regions, these percentages shifted to 39.80% for the GC and 60.20% for the AT. Remarkably, the SSC region stood apart, boasting a strikingly low GC content of just 13.84% and a correspondingly elevated AT content of 86.16%, differing from both the LSC and IR regions.

Based on the complete gene annotation, the cp genome of *S. pinguicula* contained 103 unique genes, with 20 genes duplicated within the IR regions, totaling 123 genes (Table 1). Among these, 77 were annotated as protein-coding genes, 38 as tRNA genes, and 8 as rRNA genes. Specifically, 34 were related to photosynthesis, 31 genes were associated with self-replication, and there were 6 other genes with unknown functions. In addition, among the 30 tRNA genes, 8 genes had two copies: *trnA-UGC*, *trnH-GUG*, *trnI-CAU*, *trnI-GAU*, *trnL-CAA*, *trnN-GUU*, *trnR-ACG*, and *trnV-GAC*. And, all eight rRNA genes (*rrn4.5*, *rrn5*, *rrn16*, and *rrn23*) also existed in two copies.

**Figure 3:**
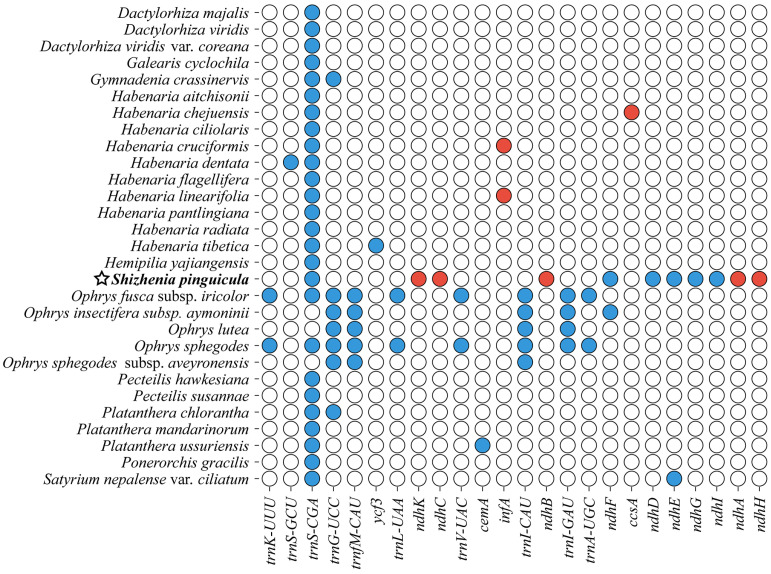
Distribution of pseudogenes and lost genes within the cp genomes of the Orchidinae species. Color blocks represent gene states, with white indicating the presence of the gene, red indicating pseudogenization, and blue indicating gene absence. The cp genome of *S. pinguicula* assembled here is marked with a star.

In the cp genome of *S. pinguicula*, a total of twelve protein-coding genes and eight tRNA genes contained introns (Table S1). Except for the *rps12*, *clpP*, and *ycf3* genes, which had two introns each, the remaining 16 genes all contained a single intron: *atpF*, *petB*, *petD*, *rpl16*, *rpoC1*, *rps16*, *rpl2* (×2), *trnK-UUU*, *trnG-UCC*, *trnL-UAA*, *trnV-UAC*, *trnI-GAU* (×2), and *trnA-UGC* (×2). The *trnK-UUU* gene possessed the largest intron, which covered the *matK* gene, a trait universally observed in green plants. Intriguingly, the NDH genes underwent significant changes, with several members, including *ndhA*, *ndhB*, *ndhC*, *ndhH*, and *ndhK*, undergoing pseudogenization, while *ndhD*, *ndhE*, *ndhF*, *ndhG*, and *ndhI* were completely lost from the cp genome (Figure 3).

### 3.2. Codon Usage Analysis

**Figure 4:**
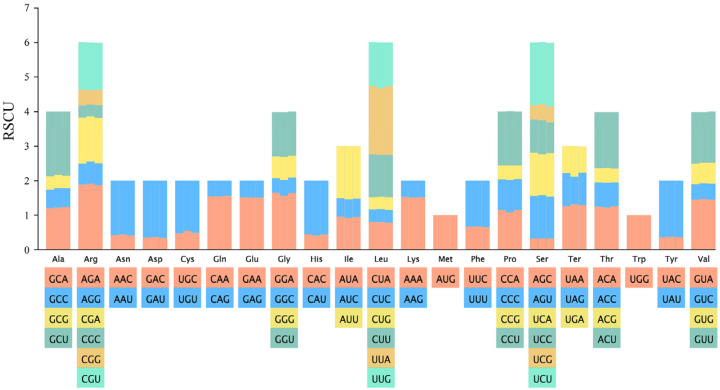
The relative synonymous codon usage (RSCU) values of each amino acid in the chloroplast genomes. The histogram represents *P. gracilis*, *S. pinguicula*, and *H. yajiangensis* from left to right.

To elucidate the codon usage patterns in the cp genome of *S. pinguicula*, we calculated RSCU values across its protein-coding gene sequences (Figure 4, Table S2). Among all codons encoding amino acids, those with high RSCU values (RSCU > 1.6) included UUA for leucine, AGA for arginine, GCU for alanine, UCU for serine, GAU for aspartic acid, UAU for tyrosine, and ACU for threonine. The codons AAA for lysine, AUU for isoleucine, and GAA for glutamic acid emerged as the most frequent, with counts of 762, 740, and 738, respectively. In contrast, UGC for cystine was the least abundant, with a count of merely 53. The codons for leucine (UUG, UUA, CUA, CUU, CUC, and CUG) dominated, reaching a total count of 1689, while the codons for cystine (TGT and TGC) were the rarest, amounting to 192. Analyzing the entire coding sequence, we identified 30 high RSCU codons (RSCU > 1), with 29 of them ending in A/U (96.67%). Conversely, among the 32 low RSCU codons (RSCU < 1), 29 of these terminated in C/G (90.63%). And, the RSCU values for UGG (encoding tryptophan) and AUG (encoding methionine) were precisely 1, revealing no discernible bias in their usage. These findings pointed to a pronounced preference for A/U-ending codons over C/G-ending ones in the *S. pinguicula* cp genome.

**Figure 5:**
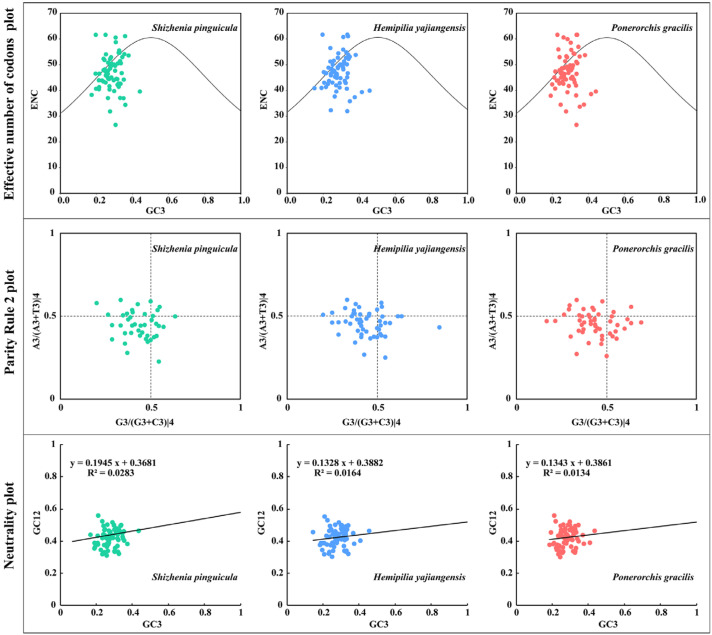
The effective number of codons (ENC), the Parity Rule 2 (PR2), and the neutrality plots for the chloroplast genomes of *S. pinguicula*, *H. yajiangensis*, and *P. gracilis*. The dots in the figure represent genes. The ENC plot shows the GC3 on the *x*-axis and the ENC on the *y*-axis. The PR2 plot shows G3/(G3 + C3)|4 on the *x*-axis and A3/(A3 + T3)|4 on the y-axis, and indicates a bias towards A/G in the first quadrant and T/C in the third quadrant, with no preference centered. The neutrality plot displays the GC3 on the *x*-axis and the GC12 (average of the GC1 and GC2) on the *y*-axis.

The average GC content in CDS of the cp genome from *S. pinguicula* was 37.32% (Table S3), with GC1 (44.94%) > GC2 (39.43%) > GC3 (27.61%). The ENC values spanned from 26.02 to 61.00, averaging at 46.21%. Neutrality plot analysis revealed that, similarly to *H. yajiangensis* and *P. gracilis*, the GC3 values ranged from 16.47% to 43.18%, and the GC12 values ranged from 31.25% to 56.10%. The regression coefficient for *S. pinguicula* was 0.1945, with a corresponding coefficient of determination (R²) of 0.0283, indicating a non-significant correlation between the GC12 and GC3 (Figure 5). In the ENC analysis, *S. pinguicula* displayed a scattered ENC distribution primarily clustering around the standard curve, with some points deviating above or below it, and a minority aligning on the curve. The results of the PR2 plot analysis showed that the gene loci of the three species was unevenly distributed across the four regions, with the highest distribution in areas where both A3/(A3 + T3)|4 and G3/(G3 + C3)|4 were less than 0.5, suggesting a preference for T > A and C > G at the third base, indicating a selective preference.

### 3.3. Selective Pressure Analysis

**Figure 6:**
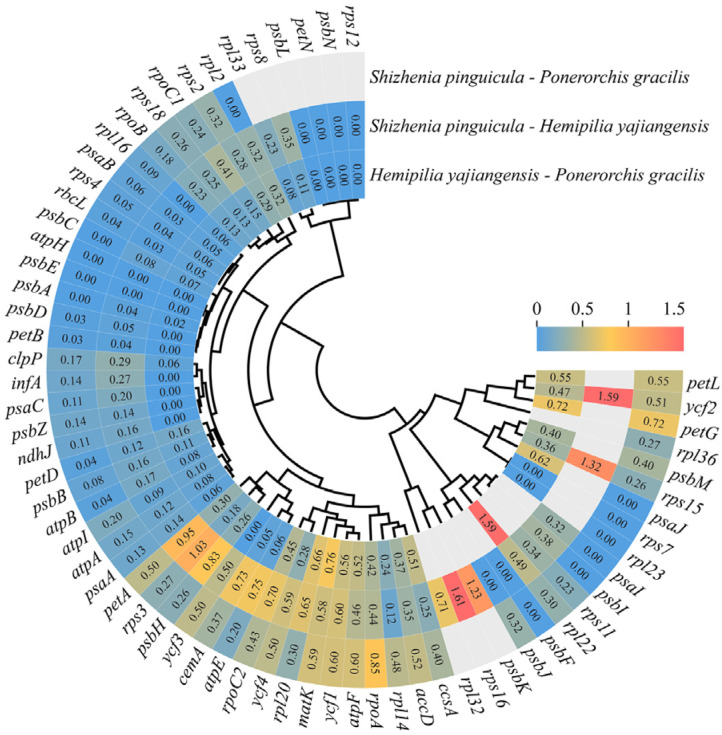
The heatmap illustrates the pairwise Ka/Ks ratios for each individual gene across the cp genomes of *S. pinguicula*, *H. yajiangensis*, and *P. gracilis*. The numerical values within the squares represent the Ka/Ks ratios, where red indicates positive selection, and yellow and blue (with Ka/Ks < 1) indicate purifying selection.

To investigate the evolutionary characteristics in the cp genome of *S. pinguicula*, the Ka/Ks ratios were calculated for 69 shared CDS genes in the cp genomes of *S. pinguicula*, *H. yajiangensis*, and *P. gracilis* (Figure 6). The majority of genes involved in photosynthesis and self-replication displayed Ka/Ks values below 1, suggesting that these genes are under negative purifying selection. Photosynthesis-related genes such as *psaB*, *psbD*, *petA*, *atpA*, *ndhJ*, and *rbcL*, as well as self-replication-related genes like *rpoC1*, *rpl14*, and *rps18*, fell into this category. Meanwhile, the Ka/Ks ratios for *rps16*, *ycf2*, *rps15*, *psbK* and *rps3* were found to be greater than 1 when comparing *S. pinguicula* and *H. yajiangensis*, but less than 1 in comparisons with other species, hinting at possible positive selection acting on these genes.

**Figure 7:**
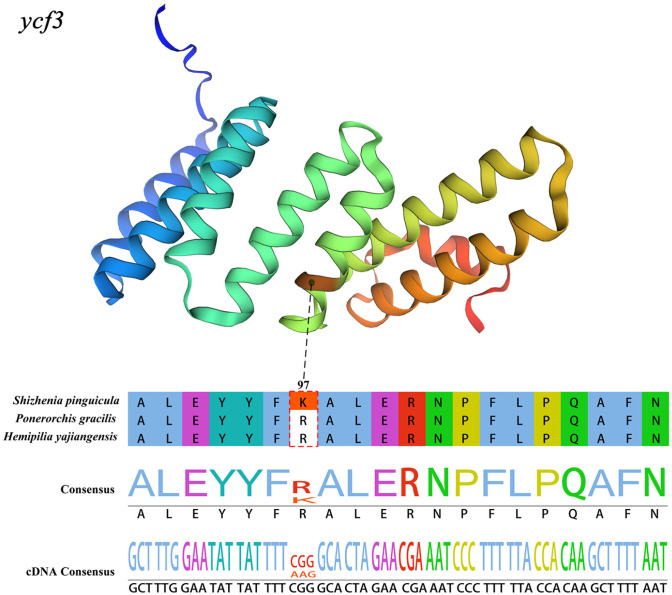
The amino acid sequence and spatial distribution of the positively selected site within the *ycf3* gene. The red box represents the site in *S. pinguicula* with the Bayes Empirical Bayes (BEB) posterior probabilities > 0.95.

To further ascertain whether it is under positive selection, we conducted a branch-site model analysis on 69 CDS genes within the cp genomes, using *S. pinguicula* as the foreground branch. Our result indicated that the 97th site of the *ycf3* gene exhibited a significant likelihood ratio test result (0.0006), with a BEB posterior probability (0.984) exceeding 0.95. Therefore, this site was identified as a positive selection site. Notably, both *H. yajiangensis* and *P. gracilis* encoded arginine at this site, whereas *S. pinguicula* encoded lysine. In terms of the protein spatial distribution of this gene, the positive selection site was located within an α-helix (Figure 7).

### 3.4. Dispersed Repeats and SSRs Analysis

**Figure 8:**
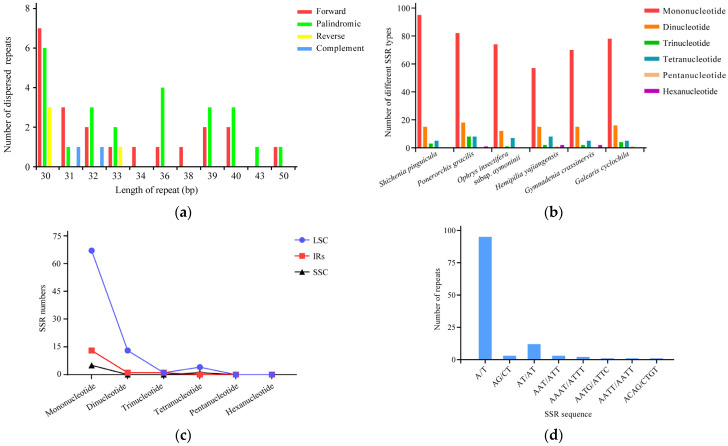
The identification of dispersed repeat sequences and simple sequence repeats (SSRs) in the cp genome of *S. pinguicula*. (**a**) The number of dispersed repeat sequences of varying lengths within the cp genome of *S. pinguicula*. (**b**) The number of SSRs in the cp genomes of *S. pinguicula* and five other Orchideae species. (**c**) The number of SSRs in the LSC, SSC, and IR regions of the cp genome of *S. pinguicula*. (**d**) The number of SSRs with different motifs. LSC: large single-copy region; SSC: small single-copy region; IRs: inverted repeat regions.

Utilizing REPuter, the cp genome of *S. pinguicula* was analyzed to identify repeat sequences exceeding 30 bp in length. A total of 51 dispersed repeat sequences were detected, categorized into 21 forward repeats, 4 reverse repeats, 24 palindromic repeats, and 2 complementary repeats (Table S4). The majority of these sequences ranged between 30 and 39 bp (84.31%), followed by 40–49 bp (11.76%), and the least frequent were those spanning 50–59 bp (3.92%) (Figure 8a).

The SSR analysis revealed that the cp genome of *S. pinguicula* contained 118 SSRs, including 95 mononucleotide repeats (80.51%), 15 dinucleotide repeats (12.72%), 3 trinucleotide repeats (2.54%), and 5 tetranucleotide repeats (4.24%), with no occurrence of pentanucleotide or higher-order repeats (Figure 8b). When examining the distribution pattern of these SSRs, on one hand, we found that 85 repeats were situated in the LSC region (72.03%), 27 in the IR regions (22.88%), and 6 in the SSC region (5.08%) (Figure 8c). On the other hand, 65 of these repeats were located in the IGS (55.08%), 35 in exons (29.67%), and 18 in introns (15.25%), indicating a predominant distribution of SSRs within the IGS (Table S5). The mononucleotide, dinucleotide, trinucleotide, and tetranucleotide SSR types in the cp genome of *S. pinguicula* were mainly composed of A/T (100%), AT/AT (80%), AAT/ATT (100%), and AAAT/ATTT (40%), respectively (Figure 8d). Overall, these findings suggested the prevalence of A/T-based mononucleotide repeats among the SSRs and a strong preference for A and T nucleotides in the SSRs of this genome.

### 3.5. Boundaries of Junction Sites Analysis

**Figure 9:**
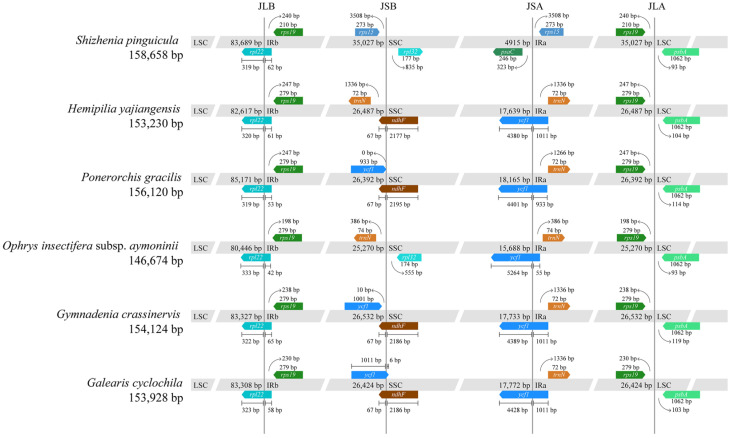
Comparison of the LSC, SSC, and IR regions’ boundaries in the cp genome of *S. pinguicula* and its five close relatives.

The comparative analysis of four boundaries across the cp genomes of the six Orchidaceous species revealed the relative conservation of the cp genome structure, despite some degree of variation in sequence length and the dynamic processes of contraction and expansion observed at the boundaries of its various partitions (Figure 9). Genes positioned at the boundaries of the cp genome included *rpl22*, *rps19*, *rps15*, *trnN*, *ycf1*, *rpl32*, *ndhF*, *psaC*, and *psbA*.

At the boundary between the IRa and LSC, all six species exhibited similarity, residing in the IGS region between the *rps19* and *psbA* genes, with minor variations in length. The boundaries in *H. yajiangensis* and *P. gracilis* were 247 bp apart from the *rps19* gene, while *S. pinguicula*, *Gy. crassinervis*, *Ga. cyclochila*, and *O. insectifera* subsp. *aymoninii* displayed distances of 240 bp, 238 bp, 230 bp, and 198 bp, respectively. With respect to the *psbA* gene, a slight difference was noted, as *S. pinguicula* and *O. insectifera* subsp. *aymoninii* maintained a 93 bp separation, contrasted with 104 bp, 114 bp, 119 bp, and 103 bp for the remaining species. Examining the LSC and IRb boundary, all six species exhibited a common localization within the *rpl22* gene, differing only subtly in the boundary position. This boundary divided the *rpl22* gene into distinct segments belonging to the LSC and IRb regions. The length within the LSC region fluctuated between 319 bp and 333 bp, whereas the length within the IRb region ranged from 42 bp to 65 bp.

Significant variations were observed in the IRb and SSC boundaries among the species. In *H. yajiangensis*, *P. gracilis*, *Gy. crassinervis*, and *Ga. cyclochila*, the IRb and SSC boundary fell within the *ndhF* gene, dividing it into IRb and SSC segments. The IRb portion remained consistent at 67 bp across these species, while the SSC portion varied slightly, with 2186 bp in *Gy. crassinervis* and *Ga. cyclochila* and 2177 bp and 2195 bp, respectively, in *H. yajiangensis* and *P. gracilis*. However, in *O. insectifera* subsp. *aymoninii*, the absence of the *ndhF* and *ycf1* genes shifted the JSB boundary to the IGS region between the *trnN* and *rpl32* genes, positioned 386 bp from the *trnN* gene and 555 bp from the *rpl32* gene. The extra copies of the *ycf1* and *rps15* genes triggered a significant expansion of the IR regions in *S. pinguicula*, extending it to 35,027 bp. Consequently, the IR regions experienced a dramatic expansion, causing an unexpected placement of the IRb and SSC boundary in the IGS region between the *rps15* and *rpl32* genes, at a distance of 3508 bp from the *rps15* gene and 177 bp from the *rpl32* gene.

Similarly, considerable variations were observed in the SSC and IRa boundaries. Except for *S. pinguicula*, the boundaries of the remaining species were positioned within the *ycf1* gene, segregating it into SSC and IRa regions. The SSC and IRa lengths were 4380 bp and 1011 bp in *H. yajiangensis*, 4401 bp and 933 bp in *P. gracilis*, 4389 bp, 1011 bp in *Gy. crassinervis*, 4428 bp and 1011 bp in *Ga. cyclochila*, and 5264 bp and 55 bp in *O. insectifera* subsp. *aymoninii*. Notably, the extensive pseudogenization and loss of the NDH genes resulted in a contraction of the SSC region to merely 4915 bp, further compressing the SSC and IRa boundary to the IGS region between the *psaC* and *rps15* genes, positioned 323 bp from the *psaC* gene and 3508 bp from the *rps15* gene.

### 3.6. Nucleotide Diversity

The mVISTA alignment demonstrated that the arrangement orders of cp genomes were consistent across species, displaying a high conservation (Figure S1). Within these genomes, non-coding regions displayed a heightened variation, in contrast to exons, introns, tRNA, and rRNA, which displayed relatively low variations. The IR regions of the cp genome showed a lower variation than the LSC and SSC regions, with the LSC region displaying the highest variation, suggesting evolutionary conservation within the IR regions.

**Figure 10:**
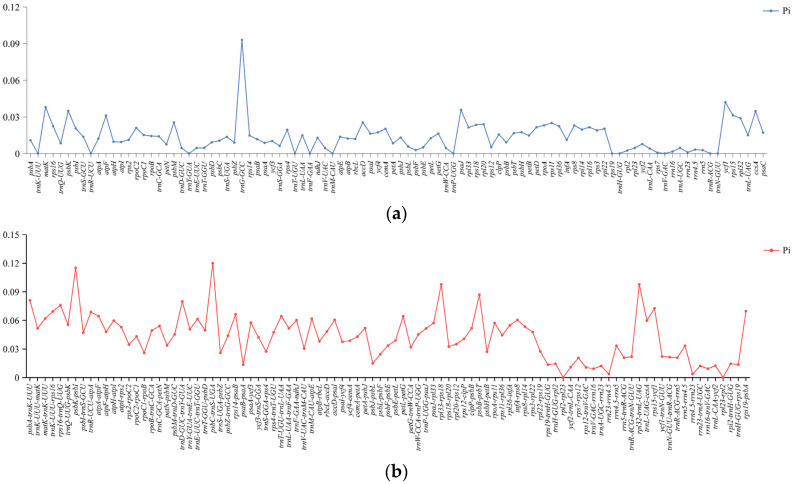
Nucleotide polymorphism (Pi) in the cp genomes of *S. pinguicula* and its five closely related species. (**a**) The Pi of the common genes in the cp genomes. (**b**) The Pi of the common intergenic spacer (IGS) regions.

Upon extracting 98 common genes and 95 common IGS sequences from the cp genomes of six species, the nucleotide polymorphism (Pi) values were calculated (Figure 10). Generally, the Pi values of the IGS regions surpassed those of the common genes. The IR regions emerged as more conserved than the LSC and SSC regions, aligning with the mVISTA analysis. Among these, we identified ten hypervariable regions (Pi > 0.07), including *psbC-trnS-UGA*, *psbK-psbI*, *rpl32-trnL-UAG*, *rpl33-rps18*, *trnG-GCC*, *psbB-psbT*, *psbA-trnK-UUU*, *trnD-GUC-trnY-GUA*, *rps16-trnQ-UUG*, and *rps15-ycf1*. These hypervariable regions offered an avenue for investigating species identification, the evolutionary process, and the adaptation mechanism.

### 3.7. Phylogenetic Relationships

**Figure 11:**
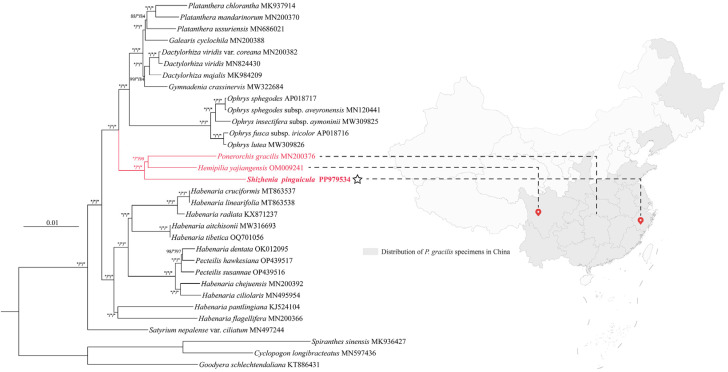
The phylogenetic relationships of the Orchideae subtribe inferred using the complete cp genome based on the ML, BI, and MP methods. The support values on the branches are presented in the order of BP_ML_/PP_BI_/BP_MP_ (“*” indicates a support value of BP = 100 or PP = 1.0). The red branches indicate the East Asian clade. The main distribution locations of the three species from the East Asian clade are marked on the map of China on the right. The outgroups were *Cyclopogon longibracteatus* (MN597436), *Goodyera schlechtendaliana* (KT886431), and *Spiranthes sinensis* (MK936427) from the Cranichideae tribe. The cp genome of *S. pinguicula* assembled here is marked with a star.

Phylogenetic trees were reconstructed using the cp genome sequences of 32 species to determine the phylogenetic relationship and generic position of *Shizhenia* (Figure 11). The phylogenetic trees based on the ML, BI and MP methods demonstrated an overall high level of support, with high resolution at the trunk. The phylogenetic trees showed that the genus *Satyrium* formed a separate clade, a sister to the remaining 28 species of the tribe Orchidinae. These 28 species further divided into two major branches. One comprised *Habenaria* and *Pecteilis*, wherein *Habenaria* did not maintain a monophyletic status and instead mixed with *Pecteilis* to form a closely related sister group. The other branch was constituted by multiple genera, with a high level of support for its monophyly. Among them, *Shizhenia* occupied the basal position within this branch, being most closely affiliated with the clade formed by *Hemipilia* and *Ponerorchis*, acting as the sister group to the remaining 13 species. Immediately adjacent to this basal clade, the genus *Ophrys* displayed the strongest monophyletic support, forming a close relationship. Within the following branch, the sister group consisting of *Dactylorhiza* and *Gymnadenia* was strongly supported, while this branch stood as a sister group to the monophyletic clade comprising *Platanthera* and *Galearis*.

## 4. Discussion

### 4.1. Chloroplast Genome Structure and Basic Characteristics

The structure of the cp genome in *S. pinguicula* was similar to that of most green plants, featuring a typical circular quadripartite structure composed of the LSC, SSC, and two IR regions [56]. Our study assembled a cp genome for *S. pinguicula* with a size of 158,658 bp, which aligned closely with those of other Orchideae species [32,33]. Only 123 genes were annotated in *S. pinguicula*, fewer than the maximum of 133 genes identified in other species [32,47]. This reduced gene count was primarily due to the extensive loss of NDH genes in *S. pinguicula*, in contrast to the minimal or no loss observed in other Orchideae species. During the evolution of cp genomes, gene loss and alterations in IR boundaries, including contractions and expansions, can introduce structural variations in genes [57,58]. Some studies have suggested that the missing NDH genes were dispensable in some Orchidaceae species, as they can be functionally replaced by homologous genes with similar functions encoded in the nuclear genome [42,59]. The loss and pseudogenization of NDH genes in the cp genome of *S. pinguicula* led to the contraction of the SSC region, further resulting in a decrease in the GC content to 13.84% in this region. Conversely, the size and number of the *ycf1* and *rps15* genes accounted for the expansion of the IR boundaries. Such unprecedented contractions and expansions of boundaries have not been observed in other Orchideae species with published cp genomes from the NCBI database [22,32,33,43,47,48,60]. Thus, sequencing the cp genomes of more species within these tribes will be necessary to provide more comprehensive information of the region-boundary variations.

### 4.2. Codon Usage Pattern and Selective Pressure

The usage bias of codons reflects the origin, mutation patterns, and evolution of species or genes, showing variations across the genomes of diverse organisms [25,27]. Our study revealed that the cp genome of *S. pinguicula* exhibited a higher frequency of usage for 30 codons (RSCU > 1), predominantly terminating in A/U, with the codon encoding leucine occupying the top spot. And, 32 codons were found to be less frequently used (RSCU < 1), largely ending in C/G, with the codon for cystine being in the lowest position. These showed that the expression levels of proteins related to leucine were higher. Despite some differences, *S. pinguicula* displayed similar codon usage preferences to both *P. gracilis* and *H. yajiangensis*, which may be related to natural selection, such as adaptation to low-altitude and moist growth environments. The results of neutrality plot analysis indicate that the codon usage bias in the cp genome of *S. pinguicula* is primarily influenced by natural selection and other factors, with a relatively minor impact from mutational pressure. In the ENC-plot analysis, the ENC for most genes was far from the standard curve, similarly suggesting that natural selection and other factors predominantly affect codon bias. The PR2-plot analysis revealed that the third position of codons in most genes shows a preference for T and C, indicating that selective pressure significantly influences the codon usage pattern. These results collectively demonstrate that selective pressure plays a prominent role in determining codon preferences.

Furthermore, the selective pressure analysis showed that the genes *rps16*, *ycf2*, *rps15*, *psbK*, and *rps3* may be under positive selection. These genes have a limited influence on the species differentiation between *P. gracilis* and *H. yajiangensis* but play a crucial role in facilitating the divergence between *S. pinguicula* and *H. yajiangensis*. *Shizhenia pinguicula* is a species primarily distributed in coastal areas such as Zhejiang Province in China, while *H. yajiangensis* is found only in Sichuan, China. In contrast, *P. gracilis* has a relatively broader distribution range (Figure 11). These genes under potential positive selection are involved in photosynthesis or self-replication, leading us to speculate that this may be an adaptation to the unique environments of mountainous coastal regions. To further identify the target sites of positive selection, we conducted a branch-site model analysis on 69 CDS genes within the cp genomes, using *S. pinguicula* as the foreground branch. We discovered a positive selection site within the *ycf3* gene, located in the α-helix of the protein space and responsible for encoding lysine. Our results indicated that the protein-coding genes of *S. pinguicula* are relatively conserved, with only the *ycf3* gene undergoing positive selection. This gene has also been observed to undergo positive selection in other plants, such as those belonging to Fagaceae, Zingiberaceae, and Leguminosae [61,62,63]. The *ycf3* gene is essential for the initial assembly of the newly synthesized subunits PsaA/B into a reaction center subcomplex [64]. These PsaA/B subunits, along with peripheral core subunits, form the photosystem I complex, which catalyzes electron transfer to reduce NADP^+^ to NADPH [65]. Finally, photosystem I and photosystem II together convert solar energy into redox energy, enabling photosynthesis.

### 4.3. Identification of Repeats and Hypervariable Sequences

The DNA of plant organelles possesses inexplicable complexity and variability [66]. The cp genome is abundant in SSRs and dispersed repeats. These complex DNA elements play a crucial role in the species identification and genetic diversity analysis of plants, due to their characteristics of high polymorphism, stability, accessibility, and codominant inheritance [67,68]. A total of 118 SSR loci were identified in the cp genome of *S. pinguicula*, with a concentration in the LSC region (72.03%), primarily composed of polyA and polyT repeats. And, mononucleotide SSRs dominated the landscape, accounting for the majority (80.51%) of all identified SSRs. The prevalence of short repeats as the main repeat type may have indicated the specificity or the differing selective pressures acting upon this species, suggesting a relatively advanced evolutionary state for *S. pinguicula* [69]. Additionally, our analysis uncovered 51 dispersed repeat sequences in *S. pinguicula*, including forward, reverse, palindromic, and complementary repeats, with forward repeats being prominent. Compared to the study of *Vietorchis* cp genome, more available dispersed repeats were discovered [60]. The SSR loci and dispersed repeat sequences identified here have potential as markers for assessing genetic diversity and informing conservation strategies for Orchideae species.

Effective hypervariable sequences, vital for species identification and phylogenetic analysis, can be screened out based on cp genome comparisons [70,71]. Employing the mVISTA and DnaSP software, we identified ten promising variable markers: *psbC-trnS-UGA*, *psbK-psbI*, *rpl32-trnL-UAG*, *rpl33-rps18*, *trnG-GCC*, *psbB-psbT*, *psbA-trnK-UUU*, *trnD-GUC-trnY-GUA*, *rps16-trnQ-UUG*, and *rps15-ycf1*. Characterized by high sequence diversity, these markers offer avenues for future phylogenetic investigations and the species identification of the tribe Orchideae. Nevertheless, the limitation of publicly available cp genome sequences for Orchideae highlights the urgent necessity to sequence and analyze more genera and species from this tribe.

### 4.4. Phylogenetic Analysis

*Shizhenia pinguicula* stands as one of the earliest species identified within genus *Amitostigma*, which was established by Schlechter in 1919 [1]. For nearly a century, it retained its status within this genus until advancements in phylogenetic research sparked disagreements among scholars over its taxonomic classification [2,3,6,7,8,16]. Jin et al. [3] merged the entire genus *Amitostigma* and *Neottianthe* into *Ponerorchis* s.l. based on their molecular phylogenetic analysis and intergeneric morphological comparisons. The phylogenetic analysis [8], which was conducted using the cp genome markers *rbcL*, *matK*, *trnL-F*, *psbA-trnH*, and *trnS-trnG*, as well as *Xdh* and nrITS, yielded similar results. Tang et al. [6] further expanded the sampling, and their study covered the N1 clade (solely consisting of *S. pinguicula*) and the N2 clade (consisting of multiple species of *Amitostigma* and *Ponerorchis*, including the type species of *Ponerorchis*). In the phylogenetic trees constructed based on nrITS and *Xdh*, *S. pinguicula* would render the *Ponerorchis* paraphyletic. Merging *Amitostigma* and its allies (*Neottianthe* Schltr., *Ponerorchis*, *Tsaiorchis* Tang and F.T.Wang, and *Hemipiliopsis*) from East Asia into *Hemipilia* s.l. is an option. However, upon expanding the sampling of *Ponerorchis*, the phylogenetic tree based on nuclear markers (ITS + Xdh) revealed that the N2 clade is dispersed into the multifurcating clade of *Ponerorchis* s.l. [7]. Thus, the classification of the East Asian clade within the subtribe Orchidinae currently remains controversial. Of course, more work is still needed to determine the division and stability of *Shizhenia*.

Recently, plant phylogenetics based on cp genomes has become increasingly popular [32,72,73]. We selected 10 species from 29 genera within the Orchidinae subtribe and constructed ML, BI, and MP trees using cp genome sequences. Our phylogenetic trees showed that *H. yajiangensis* and *P. gracilis* clustered together. In Jin’s phylogenetic tree [7], these two species are located in the widely spaced Subclade XV (combined with Yang’s results [22]) and Subclade I branches, respectively. Despite the limited availability of cp genomes in this study, *S. pinguicula* still forms a sister clade with the branch containing these two species, which is consistent with previous studies showing its basal position within the East Asia clade of the Orchidinae subtribe [6,7,8,22]. Here, we emphasize the importance of *S. pinguicula* as a basal taxonomic unit, providing the possibility for further expanding sampling and reconstructing the phylogenetic relationships among this species and its closely related species. Further research is still needed to confirm the structural reliability of the phylogenetic trees within the subtribe Orchidinae.

## 5. Conclusions

In this study, we assembled the complete cp genome of *S. pinguicula* for the first time. We then conducted a detailed analysis of this cp genome and performed comparative analyses with its closely related species. Based on these analyses, we conclude that the cp genome structure and gene content of *S. pinguicula* display certain degrees of variation compared to other species within the Orchidinae subtribe. Although the cp genome of *S. pinguicula* maintains a conservative gene order and stable GC content, a notable number of NDH genes are either pseudogenized or lost, and changes in the boundaries of the IR regions are observed, leading to an expansion of the genome. The genes *ycf3*, *ycf2*, *rps16*, *rps15*, *psbK*, and *rps3* may have undergone positive selection, potentially associated with the adaptive evolution of *S. pinguicula*. The phylogenetic analysis indicated that *H. yajiangensis* and *P. gracilis* are the closest relatives of *S. pinguicula*. Notably, we detected 118 SSRs in *S. pinguicula*, with some hotspots showing potential applications in DNA barcoding for species identification. The cp genome sequence of *S. pinguicula* reported in this study serves as an important resource for further investigations into the biology of the Orchidinae subtribe and for the formulation of conservation strategies.

## Figures and Tables

**Table 1:** Genes present in the chloroplast genome of *S. pinguicula*.

Category	Gene Group	Gene Name
RNA genes	Ribosomal RNA genes (rRNA)	*rrn4.5* (×2), *rrn5* (×2), *rrn16* (×2), *rrn23* (×2)
	Transfer RNA genes (tRNA)	*trnA-UGC ^a^* (×2), *trnC-GCA*, *trnD-GUC*, *trnE-UUC*, *trnF-GAA*, *trnG-GCC*, *trnG-UCC ^a^*, *trnH-GUG* (×2), *trnI-CAU* (×2), *trnI-GAU ^a^* (×2), *trnK-UUU ^a^*, *trnL-CAA* (×2), *trnL-UAA ^a^*, *trnL-UAG*, *trnM-CAU*, *trnN-GUU* (×2), *trnP-UGG*, *trnQ-UUG*, *trnR-ACG* (×2), *trnR-UCU*, *trnS-GCU*, *trnS-GGA*, *trnS-UGA*, *trnT-GGU*, *trnT-UGU*, *trnV-GAC* (×2), *trnV-UAC ^a^*, *trnW-CCA*, *trnY-GUA*, *trnfM-CAU*
Photosynthesis	Photosystem I	*psaA*, *psaB*, *psaC*, *psaI*, *psaJ*
	Photosystem II	*psbA*, *psbB*, *psbC*, *psbD*, *psbE*, *psbF*, *psbH*, *psbI*, *psbJ*, *psbK*, *psbL*, *psbM*, *psbN*, *psbT*, *psbZ*
	Subunit of cytochrome	*petA*, *petB ^a^*, *petD ^a^*, *petG*, *petL*, *petN*
	Subunit of synthase	*atpA*, *atpB*, *atpE*, *atpF ^a^*, *atpH*, *atpI*
	Large subunit of rubisco	*rbcL*
	NADH dehydrogenase	*ndhJ*
Self-replication	Small subunit of ribosome	*rps2*, *rps3*, *rps4*, *rps7* (×2), *rps8*, *rps11*, *rps12 ^b^* (×2), *rps14*, *rps15* (×2), *rps16 ^a^*, *rps18*, *rps19* (×2)
	Large subunit of ribosome	*rpl2 ^a^* (×2), *rpl14*, *rpl16 ^a^*, *rpl20*, *rpl22*, *rpl23* (×2), *rpl32*, *rpl33*, *rpl36*
	DNA-dependent RNA polymerase	*rpoA*, *rpoB*, *rpoC1 ^a^*, *rpoC2*
Other genes	Maturase	*matK*
	Chloroplast envelope membrane protein	*cemA*
	Translational initiation factor	*infA*
	Subunit acetyl-CoA carboxylase	*accD*
	C-type cytochrome synthesis	*ccsA*
	ATP-dependent protease subunit P	*clpP ^b^*
Unknown	Conserved open reading frames	*ycf1* (×2), *ycf2* (×2), *ycf3 ^b^*, *ycf4*

*Gene ^a^*: gene with one intron; *Gene ^b^*: gene with two introns; *Gene* (×2): number of copies of multi-copy genes.

## Data Availability

The chloroplast genome sequence supporting this study has been uploaded to GenBank (National Center for Biotechnology Information) with the accession number PP979534. The BioProject, BioSample, and SRA numbers are PRJNA1131101, SAMN42254178, and SRR29687138, respectively. Additional materials supporting the results of this article are included in the Supplementary Files.

## Supplementary Materials

The following supporting information can be downloaded at: https://www.mdpi.com/article/10.3390/genes15111488/s1, Table S1: Genes with introns in the *S. pinguicula* chloroplast genome and the length of introns and exons; Table S2: The relative synonymous codon usage of the *S. pinguicula* chloroplast genome; Table S3: The GC content of the 69 CDS genes within the chloroplast genome of *S. pinguicula*; Table S4: Dispersed repeat sequence statistics data and distribution; Table S5: SSRs in the chloroplast genome of *S. pinguicula*; Figure S1: Sequence similarity between *S. pinguicula* and its five closely related species was analyzed using mVISTA with the cp genome of *H. yajiangensis* as the reference genome; Figure S2: The coverage depth of the chloroplast genome of *S. pinguicula*.
