# Supplementary material for: Characterization and Phylogenetic Analysis of the First Complete Chloroplast Genome of Shizhenia pinguicula (Orchidaceae: Orchideae)
Source: Genes (Basel). 2024 Nov 20;15(11):1488. doi: 10.3390/genes15111488 (PMC11593919; doi:10.3390/genes15111488)
Supplement: Supplementary file 1 [file genes-15-01488-s001.zip › Caption of Supplementary Materials.pdf]

Table S1: Genes with introns in the *Shizhenia pinguicula* chloroplast genome and the length of introns and exons;

Table S2: The relative synonymous codon usage of the *Shizhenia pinguicula* chloroplast genome;

Table S3: The GC content of the 69 CDS genes within the chloroplast genome of *Shizhenia pinguicula*;

Table S4: Dispersed repeat sequence statistics data and distribution;

Table S5: SSRs in the chloroplast genome of *Shizhenia pinguicula*;

Figure S1: Sequence similarity between *Shizhenia pinguicula* and its five closely related species was analyzed using mVISTA with the chloroplast genome of *Hemipilia yajiangensis* as the reference genome. The horizontal axis represents the gene position of the reference sequence, while the vertical axis indicates the similarity ranging from 50% to 100%. Exons, t/rRNAs, and conserved noncoding sequences (CNS) are represented by different colors in the lower left corner.

Figure S2: The coverage depth of the chloroplast genome of *Shizhenia pinguicula*. The horizontal axis represents the base of the chloroplast genome, while the vertical axis indicates the sequencing depth corresponding to that base.
