# Supplementary material for: Characterization and Phylogenetic Analysis of the First Complete Chloroplast Genome of Shizhenia pinguicula (Orchidaceae: Orchideae)
Source: Genes (Basel). 2024 Nov 20;15(11):1488. doi: 10.3390/genes15111488 (PMC11593919; doi:10.3390/genes15111488)
Supplement: Supplementary file 1 [file genes-15-01488-s001.zip › Table_Supplementary_Materials.pdf]

Table S1. Genes with introns in the *S. pinguicula* chloroplast genome and the length of introns and exons.

Gene	Location	Exon I (bp)	Intron I (bp)	Exon II (bp)	Intron II (bp)	Exon III (bp)
atpF	LSC	167	880	379	-	-
clpP	LSC	71	994	294	683	250
petB	LSC	6	739	642	-	-
petD	LSC	8	822	475	-	-
rpl16	LSC	9	1301	399	-	-
rpoC1	LSC	430	761	1652	-	-
rps16	LSC	40	1053	215	-	-
ycf3	LSC	124	698	230	767	153
trnK-UUU	LSC	37	2919	35	-	-
trnG-UCC	LSC	23	687	49	-	-
trnL-UAA	LSC	35	633	50	-	-
trnV-UAC	LSC	39	594	37	-	-
rpl2 (×2)	IR	385	664	431	-	-
*rps12** (×2)	IR	114	-	26	542	232
trnI-GAU (×2)	IR	42	939	35	-	-
trnA-UGC (×2)	IR	38	797	35	-	-

*rps12**: the *rps12* gene is a trans-spliced gene, with its 5' end situated within the LSC region and a pair of 3' ends positioned within the IR regions. *Gene* (×2): number of copies of multi-copy genes.

Table S2. The relative synonymous codon usage of the *S. pinguicula* chloroplast genome.

Codon	Amino Acid	RSCU	Codon	Amino Acid	RSCU
UAA	Ter	1.32	AUG	Met	1
UAG	Ter	0.8	AAC	Asn	0.44
UGA	Ter	0.88	AAU	Asn	1.56
GCA	Ala	1.22	CCA	Pro	1.08
GCC	Ala	0.56	CCC	Pro	0.94
GCG	Ala	0.39	CCG	Pro	0.42
GCU	Ala	1.83	CCU	Pro	1.57
UGC	Cys	0.55	CAA	Gin	1.55
UGU	Cys	1.45	CAG	Gin	0.45
GAC	Asp	0.37	AGA	Arg	1.91
GAU	Asp	1.63	AGG	Arg	0.65
GAA	Glu	1.51	CGA	Arg	1.3
GAG	Glu	0.49	CGC	Arg	0.35
UUC	Phe	0.68	CGG	Arg	0.41
UUU	Phe	1.32	CGU	Arg	1.38
GGA	Gly	1.56	AGC	Ser	0.33
GGC	Gly	0.46	AGU	Ser	1.25
GGG	Gly	0.67	UCA	Ser	1.19
GGU	Gly	1.3	UCC	Ser	0.98
CAC	His	0.41	UCG	Ser	0.47
CAU	His	1.59	UCU	Ser	1.79
AUA	Ile	0.92	ACA	Thr	1.22
AUC	Ile	0.54	ACC	Thr	0.72
AUU	Ile	1.54	ACG	Thr	0.43
AAA	Lys	1.51	ACU	Thr	1.62
AAG	Lys	0.49	GUA	Val	1.47
CUA	Leu	0.8	GUC	Val	0.45
CUC	Leu	0.38	GUG	Val	0.6
CUG	Leu	0.36	GUU	Val	1.47
CUU	Leu	1.21	UGG	Trp	1
UUA	Leu	1.93	UAC	Tyr	0.37
UUG	Leu	1.32	UAU	Tyr	1.63

Table S3. The GC content of the 69 CDS genes within the chloroplast genome of *S. pinguicula*.

#Id	Gene Name	GC all	GC1	GC2	GC3	G1	G2	G3	C1	C2	C3
1	*accD*	0.3299	0.3794	0.3546	0.2557	0.2557	0.1897	0.1361	0.1237	0.1649	0.1196
2	*atpA*	0.3944	0.5413	0.3996	0.2421	0.3583	0.1516	0.1437	0.1831	0.2480	0.0984
3	*atpB*	0.4238	0.5509	0.4112	0.3094	0.3713	0.1637	0.1437	0.1796	0.2475	0.1657
4	*atpE*	0.3975	0.5037	0.4148	0.2741	0.3407	0.1926	0.1556	0.1630	0.2222	0.1185
5	*atpF*	0.3535	0.4451	0.3516	0.2637	0.2912	0.2033	0.1648	0.1538	0.1484	0.0989
6	*atpH*	0.4431	0.6341	0.4878	0.2073	0.4634	0.1707	0.1585	0.1707	0.3171	0.0488
7	*atpI*	0.3683	0.4798	0.3710	0.2540	0.2984	0.1411	0.1371	0.1815	0.2298	0.1169
8	*ccsA*	0.3139	0.3221	0.3589	0.2607	0.1810	0.1564	0.1472	0.1411	0.2025	0.1135
9	*cemA*	0.3289	0.3833	0.2863	0.3172	0.2070	0.1278	0.1806	0.1762	0.1586	0.1366
10	*clpP*	0.4146	0.5854	0.3610	0.2976	0.3902	0.1610	0.1415	0.1951	0.2000	0.1561
11	*infA*	0.3632	0.4615	0.3590	0.2692	0.2308	0.2564	0.1282	0.2308	0.1026	0.1410
12	*matK*	0.3027	0.3728	0.2925	0.2428	0.1644	0.1300	0.1300	0.2084	0.1625	0.1128
13	*ndhJ*	0.3836	0.4654	0.3711	0.3145	0.2830	0.2013	0.1635	0.1824	0.1698	0.1509
14	*petA*	0.3873	0.5265	0.3583	0.2773	0.3178	0.1526	0.1589	0.2087	0.2056	0.1184
15	*petB*	0.3997	0.4722	0.4120	0.3148	0.2917	0.1806	0.1944	0.1806	0.2315	0.1204
16	*petD*	0.3582	0.4845	0.3851	0.2050	0.3043	0.1304	0.1118	0.1801	0.2547	0.0932
17	*petG*	0.3421	0.4737	0.2895	0.2632	0.2895	0.1579	0.1053	0.1842	0.1316	0.1579
18	*petL*	0.3229	0.3750	0.4063	0.1875	0.1563	0.1250	0.1250	0.2188	0.2813	0.0625
19	*petN*	0.4000	0.5000	0.4333	0.2667	0.3667	0.2333	0.2667	0.1333	0.2000	0.0000
20	*psaA*	0.4230	0.5140	0.4328	0.3222	0.3049	0.1984	0.1465	0.2091	0.2344	0.1758
21	*psaB*	0.4122	0.4816	0.4286	0.3265	0.2830	0.1946	0.1769	0.1986	0.2340	0.1497
22	*psaC*	0.3984	0.4268	0.5244	0.2439	0.2927	0.2683	0.1585	0.1341	0.2561	0.0854
23	*psaI*	0.3243	0.4054	0.2703	0.2973	0.2432	0.0541	0.1351	0.1622	0.2162	0.1622
24	*psaJ*	0.3396	0.3208	0.3962	0.3019	0.1698	0.1132	0.1887	0.1509	0.2830	0.1132
25	*psbA*	0.4200	0.4944	0.4407	0.3249	0.3277	0.2034	0.1328	0.1667	0.2373	0.1921
26	*psbB*	0.4316	0.5383	0.4617	0.2947	0.3694	0.2377	0.1631	0.1690	0.2240	0.1316
27	*psbC*	0.4409	0.5316	0.4599	0.3312	0.3523	0.2300	0.1603	0.1793	0.2300	0.1709
28	*psbD*	0.4209	0.5198	0.4350	0.3079	0.3333	0.1949	0.1469	0.1864	0.2401	0.1610
29	*psbE*	0.3889	0.4286	0.4643	0.2738	0.2619	0.2500	0.1310	0.1667	0.2143	0.1429
30	*psbF*	0.4250	0.4750	0.4500	0.3500	0.2500	0.1500	0.2000	0.2250	0.3000	0.1500
31	*psbH*	0.3739	0.4054	0.4730	0.2432	0.2838	0.1892	0.1892	0.1216	0.2838	0.0541
32	*psbI*	0.3874	0.4865	0.3243	0.3514	0.2703	0.1622	0.1081	0.2162	0.1622	0.2432
33	*psbJ*	0.4146	0.3902	0.5122	0.3415	0.2683	0.2439	0.1707	0.1220	0.2683	0.1707
34	*psbK*	0.3226	0.3065	0.3387	0.3226	0.1613	0.1290	0.0968	0.1452	0.2097	0.2258
35	*psbL*	0.2906	0.3590	0.2821	0.2308	0.1795	0.1282	0.1026	0.1795	0.1538	0.1282
36	*psbM*	0.3524	0.4571	0.2857	0.3143	0.2857	0.0286	0.1143	0.1714	0.2571	0.2000
37	*psbN*	0.4545	0.5000	0.4318	0.4318	0.3409	0.1364	0.1136	0.1591	0.2955	0.3182
38	*psbT*	0.3056	0.3611	0.3333	0.2222	0.2222	0.0833	0.1111	0.1389	0.2500	0.1111
39	*psbZ*	0.3228	0.3651	0.3968	0.2063	0.2698	0.1111	0.0794	0.0952	0.2857	0.1270
40	*rbcL*	0.4330	0.5732	0.4309	0.2948	0.3835	0.2082	0.1464	0.1897	0.2227	0.1485
41	*rpl14*	0.3740	0.5285	0.3577	0.2358	0.3496	0.1707	0.1301	0.1789	0.1870	0.1057
42	*rpl16*	0.4412	0.5000	0.5368	0.2868	0.3015	0.2868	0.1544	0.1985	0.2500	0.1324
43	*rpl2*	0.4461	0.5110	0.4926	0.3346	0.3235	0.2868	0.1581	0.1875	0.2059	0.1765
44	*rpl20*	0.3500	0.3667	0.4167	0.2667	0.1500	0.2417	0.1500	0.2167	0.1750	0.1167
45	*rpl22*	0.3281	0.4252	0.3543	0.2047	0.2835	0.1496	0.1496	0.1417	0.2047	0.0551
46	*rpl23*	0.3794	0.4149	0.4043	0.3191	0.2234	0.2128	0.2021	0.1915	0.1915	0.1170

47	*rpl32*	0.2994	0.2881	0.4237	0.1864	0.1864	0.1356	0.1186	0.1017	0.2881	0.0678
48	*rpl33*	0.3234	0.2985	0.3731	0.2985	0.1940	0.2388	0.1791	0.1045	0.1343	0.1194
49	*rpl36*	0.3772	0.4211	0.4211	0.2895	0.1579	0.3421	0.2105	0.2632	0.0789	0.0789
50	*rpoA*	0.3414	0.4277	0.3404	0.2560	0.2620	0.1566	0.1295	0.1657	0.1837	0.1265
51	*rpoB*	0.3781	0.4902	0.3774	0.2665	0.2973	0.1827	0.1696	0.1929	0.1948	0.0969
52	*rpoC1*	0.3881	0.4986	0.3775	0.2882	0.2925	0.1931	0.1643	0.2061	0.1844	0.1239
53	*rpoC2*	0.3634	0.4566	0.3649	0.2688	0.2688	0.1806	0.1467	0.1879	0.1842	0.1221
54	*rps11*	0.4269	0.5468	0.5036	0.2302	0.3453	0.2590	0.1295	0.2014	0.2446	0.1007
55	*rps12*	0.4220	0.5242	0.4839	0.2581	0.2903	0.2419	0.1048	0.2339	0.2419	0.1532
56	*rps14*	0.4059	0.4356	0.4752	0.3069	0.1881	0.2871	0.1980	0.2475	0.1881	0.1089
57	*rps15*	0.3004	0.3956	0.2637	0.2418	0.1868	0.1538	0.1648	0.2088	0.1099	0.0769
58	*rps16*	0.3490	0.4941	0.3882	0.1647	0.2706	0.2235	0.0706	0.2235	0.1647	0.0941
59	*rps18*	0.3399	0.3529	0.4118	0.2549	0.1275	0.2157	0.1667	0.2255	0.1961	0.0882
60	*rps19*	0.4000	0.4429	0.4143	0.3429	0.2571	0.1714	0.2000	0.1857	0.2429	0.1429
61	*rps2*	0.3643	0.4346	0.4051	0.2532	0.2954	0.1899	0.1646	0.1392	0.2152	0.0886
62	*rps3*	0.3318	0.4213	0.3333	0.2407	0.2546	0.1898	0.1204	0.1667	0.1435	0.1204
63	*rps4*	0.3762	0.4703	0.3861	0.2723	0.2129	0.2178	0.1535	0.2574	0.1683	0.1188
64	*rps7*	0.4103	0.5385	0.4551	0.2372	0.3205	0.2051	0.1282	0.2179	0.2500	0.1090
65	*rps8*	0.3056	0.3712	0.3561	0.1894	0.2197	0.2197	0.1061	0.1515	0.1364	0.0833
66	*ycf1*	0.2933	0.3539	0.2710	0.2549	0.1953	0.1263	0.1336	0.1586	0.1447	0.1213
67	*ycf2*	0.3792	0.4169	0.3513	0.3693	0.2164	0.1582	0.2045	0.2005	0.1930	0.1648
68	*ycf3*	0.3570	0.4556	0.3846	0.2308	0.2840	0.1479	0.1420	0.1716	0.2367	0.0888
69	*ycf4*	0.3928	0.4270	0.4054	0.3459	0.2432	0.2432	0.1892	0.1838	0.1622	0.1568

GC1-GC3 represent the GC content at the first, second, and third codon positions, respectively. G1-G3 denote the guanine (G) content at the first, second, and third codon positions, while C1-C3 represent the cytosine (C) content at the first, second, and third codon positions.

Table S4. Dispersed repeat sequence statistics data and distribution.

#Id	LengthI	StartI	Type	LengthII	StartII	Hamming distance	E-value
1	30	6516	F	30	70,914	-2	2.40E-05
2	30	8051	P	30	44,340	-2	2.40E-05
3	30	37,943	F	30	40,167	-2	2.40E-05
4	30	81,455	P	30	81,455	-2	2.40E-05
5	30	3431	P	30	3462	-3	6.73E-04
6	30	4377	R	30	14,842	-3	6.73E-04
7	30	4383	P	30	70,922	-3	6.73E-04
8	30	8050	F	30	34,890	-3	6.73E-04
9	30	9429	F	30	35,698	-3	6.73E-04
10	30	14,839	R	30	43,534	-3	6.73E-04
11	30	14,839	P	30	70,918	-3	6.73E-04
12	30	27,466	R	30	68,796	-3	6.73E-04
13	30	27,473	F	30	82,549	-3	6.73E-04
14	30	42,926	F	30	98,974	-3	6.73E-04
15	30	42,926	P	30	143,343	-3	6.73E-04
16	30	70,858	F	30	70,881	-3	6.73E-04
17	31	4389	C	31	70,922	-1	1.43E-07
18	31	53,727	F	31	53,750	-2	6.42E-06
19	31	3404	F	31	28,329	-3	1.86E-04
20	31	4387	P	31	53,853	-3	1.86E-04
21	31	81,335	F	31	81,353	-3	1.86E-04
22	32	51,314	P	32	51,314	0	3.84E-10
23	32	45,960	P	32	45,960	-2	1.71E-06
24	32	65,958	F	32	65,981	-2	1.71E-06
25	32	70,930	F	32	70,978	-2	1.71E-06
26	32	34,887	P	32	44,342	-3	5.14E-05
27	32	81,460	C	32	81,461	-3	5.14E-05
28	33	65,002	P	33	65,002	-3	1.41E-05
29	33	73,282	F	33	73,284	-3	1.41E-05
30	33	77,309	P	33	77,309	-3	1.41E-05
31	33	81,339	R	33	81,351	-3	1.41E-05
32	34	6500	F	34	70,898	-3	3.88E-06
33	36	117,108	P	36	117,108	0	1.50E-12
34	36	117,108	F	36	125,203	0	1.50E-12
35	36	125,203	P	36	125,203	0	1.50E-12
36	36	31,873	P	36	31,873	-2	8.50E-09
37	36	68,877	P	36	68,877	-2	8.50E-09
38	38	81,344	F	38	81,851	-3	2.13E-08
39	39	42,914	F	39	98,962	-2	1.56E-10
40	39	42,914	P	39	143,346	-2	1.56E-10
41	39	114,733	P	39	114,733	-3	5.78E-09
42	39	114,733	F	39	127,575	-3	5.78E-09
43	39	127,575	P	39	127,575	-3	5.78E-09
44	40	1297	P	40	1297	-2	4.11E-11
45	40	91,520	F	40	91,544	-3	1.56E-09
46	40	91,520	P	40	150,763	-3	1.56E-09
47	40	91,544	P	40	150,787	-3	1.56E-09

48	40	150,763	F	40	150,787	-3	1.56E-09
49	43	2	P	43	2	-1	1.18E-14
50	50	29,061	P	50	29,061	0	5.58E-21
51	50	70,936	F	50	70,960	0	5.58E-21

Table S5. SSRs in the chloroplast genome of *S. pinguicula*.

cpSSR ID	Repeat Motif	Length (bp)	SSR Start Position	SSR Stop Position	SSR Location	Location Type
1	T	12	1336	1347	*psbA-trnK-UUU_2*	IGS
2	A	12	3869	3880	*matK-trnK-UUU_1*	Intron
3	A	11	4384	4394	*trnK-UUU_1-rps16_2*	IGS
4	A	12	4396	4407	*trnK-UUU_1-rps16_2*	IGS
5	AT	14	6500	6513	*rps16_1-trnQ-UUG*	IGS
6	T	11	6536	6546	*rps16_1-trnQ-UUG*	IGS
7	A	10	6641	6650	*rps16_1-trnQ-UUG*	IGS
8	A	13	7077	7089	*trnQ-UUG-psbK*	IGS
9	TA	10	7589	7598	*psbK-psbI*	IGS
10	T	10	8563	8572	*trnS-GCU-trnG-UCC_1*	IGS
11	T	20	8771	8790	*trnG-UCC_1-trnG-UCC_2*	Intron
12	GTCT	12	10,778	10,789	*atpA*	Gene
13	A	15	12,074	12,088	*atpF_2-atpF_1*	Intron
14	A	10	12,242	12,251	*atpF_2-atpF_1*	Intron
15	A	13	13,187	13,199	*atpH-atpI*	IGS
16	T	14	13,300	13,313	*atpH-atpI*	IGS
17	A	20	14,848	14,867	*atpI-rps2*	IGS
18	T	10	15,665	15,674	*rps2-rpoC2*	IGS
19	T	12	17,701	17,712	*rpoC2*	Gene
20	T	10	17,768	17,777	*rpoC2*	Gene
21	T	11	17,874	17,884	*rpoC2*	Gene
22	AT	10	19,262	19,271	*rpoC2*	Gene
23	T	12	26,779	26,790	*rpoB-trnC-GCA*	IGS
24	A	14	27,474	27,487	*rpoB-trnC-GCA*	IGS
25	T	10	28,377	28,386	*trnC-GCA-petN*	IGS
26	A	11	28,734	28,744	*petN-psbM*	IGS
27	TA	12	29,081	29,092	*petN-psbM*	IGS
28	AT	10	31,180	31,189	*trnE-UUC-trnT-GGU*	IGS
29	A	10	31,383	31,392	*trnT-GGU-psbD*	IGS
30	T	11	36,362	36,372	*rps14*	Gene
31	T	11	36,540	36,550	*rps14-psaB*	IGS
32	A	10	36,560	36,569	*rps14-psaB*	IGS
33	T	10	41,462	41,471	*psaA-ycf3_3*	IGS
34	A	12	41,697	41,708	*psaA-ycf3_3*	IGS
35	A	13	43,535	43,547	*ycf3_2-ycf3_1*	Intron
36	T	11	45,713	45,723	*trnT-UGU-trnL-UAA_1*	IGS
37	A	10	45,893	45,902	*trnT-UGU-trnL-UAA_1*	IGS
38	T	11	46,030	46,040	*trnT-UGU-trnL-UAA_1*	IGS
39	A	10	46,376	46,385	*trnT-UGU-trnL-UAA_1*	IGS
40	T	13	46,839	46,851	*trnL-UAA_1-trnL-UAA_2*	Intron
41	T	12	50,177	50,188	*ndhJ-trnV-UAC_2*	IGS
42	T	11	50,340	50,350	*ndhJ-trnV-UAC_2*	IGS
43	A	11	50,975	50,985	*trnV-UAC_2-trnV-UAC_1*	Intron
44	T	12	53,569	53,580	*atpB-rbcL*	IGS
45	TAT	21	53,769	53,789	*atpB-rbcL*	IGS
46	T	12	53,865	53,876	*atpB-rbcL*	IGS

47	T	10	56,122	56,131	*rbcL-accD*	IGS
48	AATA	12	59,684	59,695	*ycf4-cemA*	IGS
49	AATG	12	60,772	60,783	*cemA*	Gene
50	T	11	62,098	62,108	*petA-psbJ*	IGS
51	T	14	64,589	64,602	*petG-trnW-CCA*	IGS
52	AT	10	65,092	65,101	*trnP-UGG-psaJ*	IGS
53	A	11	65,321	65,331	*trnP-UGG-psaJ*	IGS
54	T	11	65,960	65,970	*psaJ-rpl33*	IGS
55	A	10	66,419	66,428	*rpl33-rps18*	IGS
56	T	10	68,172	68,181	*rpl20-rps12_1*	IGS
57	A	15	68,805	68,819	*clpP_3-clpP_2*	Intron
58	A	18	68,905	68,922	*clpP_3-clpP_2*	Intron
59	ATAA	12	69,575	69,586	*clpP_2*	Gene
60	A	11	69,775	69,785	*clpP_2-clpP_1*	Intron
61	T	10	69,813	69,822	*clpP_2-clpP_1*	Intron
62	T	12	70,072	70,083	*clpP_2-clpP_1*	Intron
63	TA	10	70,876	70,885	*clpP_1-psbB*	IGS
64	TA	10	70,899	70,908	*clpP_1-psbB*	IGS
65	TA	12	70,911	70,922	*clpP_1-psbB*	IGS
66	T	12	70,929	70,940	*clpP_1-psbB*	IGS
67	AT	10	71,014	71,023	*clpP_1-psbB*	IGS
68	T	11	73,036	73,046	*psbB-psbT*	IGS
69	TA	16	73,288	73,303	*psbB-psbT*	IGS
70	GA	14	73,304	73,317	*psbB-psbT*	IGS
71	T	11	73,779	73,789	*psbN*	Gene
72	A	10	74,474	74,483	*petB_1-petB_2*	Intron
73	A	15	74,717	74,731	*petB_1-petB_2*	Intron
74	T	10	76,131	76,140	*petD_1-petD_2*	Intron
75	T	13	79,010	79,022	*rps11-rpl36*	IGS
76	T	10	80,112	80,121	*rps8-rpl14*	IGS
77	T	10	80,709	80,718	*rpl14-rpl16_2*	IGS
78	T	10	81,299	81,308	*rpl16_2-rpl16_1*	Intron
79	AT	20	81,461	81,480	*rpl16_2-rpl16_1*	Intron
80	T	10	81,997	82,006	*rpl16_2-rpl16_1*	Intron
81	T	12	82,379	82,390	*rpl16_2-rpl16_1*	Intron
82	T	11	82,535	82,545	*rpl16_1-rps3*	IGS
83	A	12	82,552	82,563	*rpl16_1-rps3*	IGS
84	T	12	83,142	83,153	*rps3*	Gene
85	T	10	83,303	83,312	*rps3-rpl22*	IGS
86	T	12	84,222	84,233	*rps19-trnH-GUG*	IGS
87	GA	10	89,765	89,774	*ycf2*	Gene
88	T	12	110,312	110,323	*ycf1*	Gene
89	A	10	110,469	110,478	*ycf1*	Gene
90	A	15	110,585	110,599	*ycf1*	Gene
91	A	11	111,734	111,744	*ycf1*	Gene
92	T	10	111,823	111,832	*ycf1*	Gene
93	A	11	111,939	111,949	*ycf1*	Gene
94	TAA	12	111,981	111,992	*ycf1*	Gene
95	T	10	112,537	112,546	*ycf1*	Gene

96	A	12	113,603	113,614	*ycf1*	Gene
97	A	10	113,743	113,752	*ycf1*	Gene
98	A	10	118,832	118,841	*rps15-rpl32*	IGS
99	T	10	119,490	119,499	*rps15-rpl32*	IGS
100	A	10	119,612	119,621	*rpl32*	Gene
101	A	10	120,091	120,100	*rpl32-trnL-UAG*	IGS
102	A	10	120,338	120,347	*rpl32-trnL-UAG*	IGS
103	T	10	121,300	121,309	*ccsA*	Gene
104	TAAT	12	121,763	121,774	*ccsA*	Gene
105	T	13	121,944	121,956	*ccsA-psaC*	IGS
106	A	11	122,718	122,728	*ccsA-psaC*	IGS
107	T	10	128,596	128,605	*ycf1*	Gene
108	T	12	128,734	128,745	*ycf1*	Gene
109	A	10	129,802	129,811	*ycf1*	Gene
110	TTA	12	130,356	130,367	*ycf1*	Gene
111	T	11	130,399	130,409	*ycf1*	Gene
112	A	10	130,516	130,525	*ycf1*	Gene
113	T	11	130,604	130,614	*ycf1*	Gene
114	T	15	131,749	131,763	*ycf1*	Gene
115	T	10	131,870	131,879	*ycf1*	Gene
116	A	12	132,025	132,036	*ycf1*	Gene
117	TC	10	152,574	152,583	*ycf2*	Gene
118	A	12	158,115	158,126	*trnH-GUG-rps19*	IGS
