# Supplementary figures and images for: Characterization and Phylogenetic Analysis of the First Complete Chloroplast Genome of Shizhenia pinguicula (Orchidaceae: Orchideae)
Source: Genes (Basel). 2024 Nov 20;15(11):1488. doi: 10.3390/genes15111488 (PMC11593919; doi:10.3390/genes15111488)

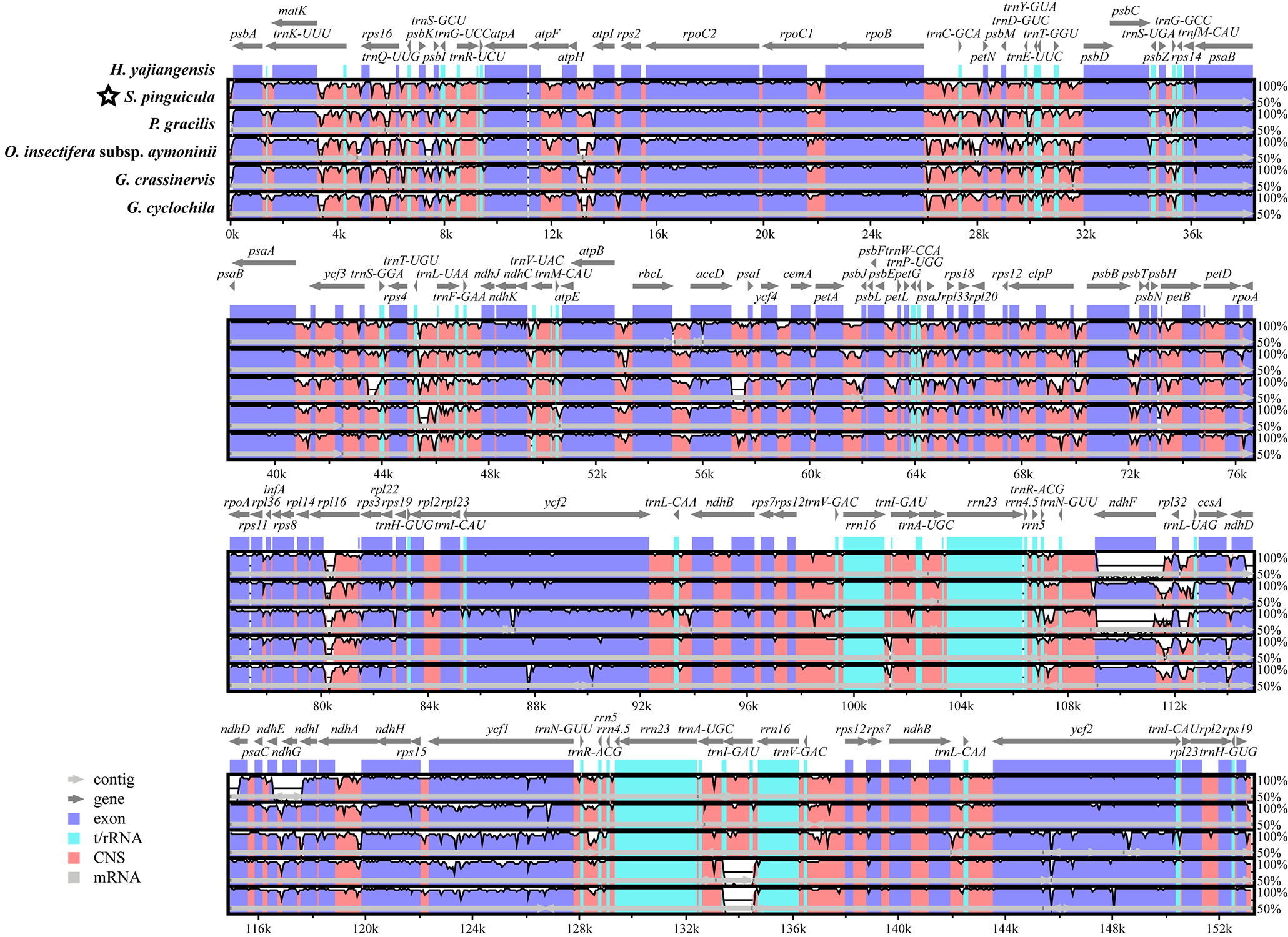

Supplement: Supplementary file 1 [file genes-15-01488-s001.zip › Figure S1.tif]

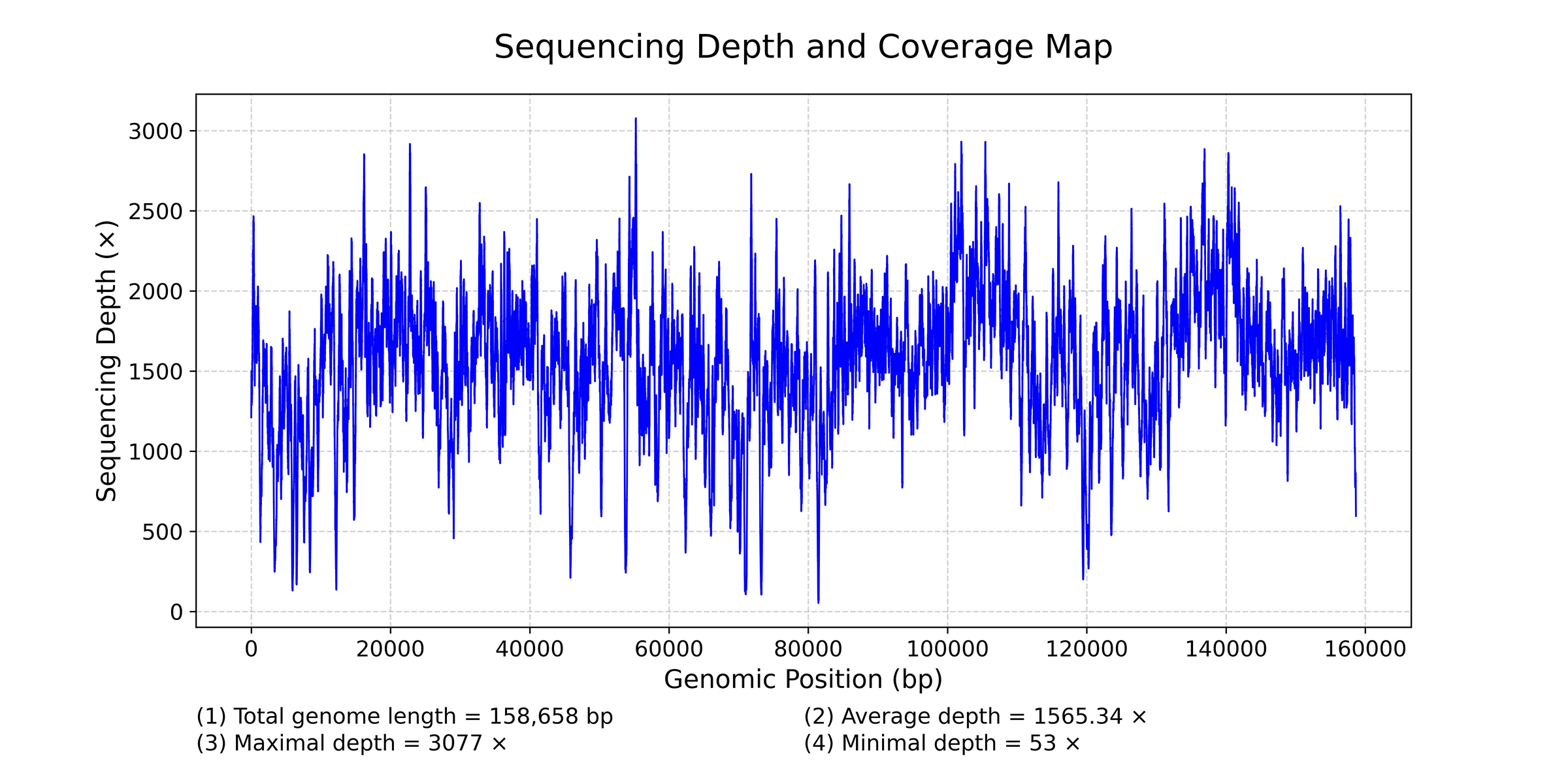

Supplement: Supplementary file 1 [file genes-15-01488-s001.zip › Figure S2.tif]
